# Resistin in cardiac diseases: from molecular mechanisms to clinical implications

**DOI:** 10.3389/fendo.2025.1708332

**Published:** 2025-12-03

**Authors:** Yicheng Ling, Baixue Han, Tianxiang Gu, Xuan Jiang

**Affiliations:** The First Hospital of China Medical University, Shenyang, China

**Keywords:** resistin, cardiac diseases, atherosclerosis, heart failure, prognostic value

## Abstract

Resistin, a cysteine-rich adipokine, exhibits significant species-specific divergence in its cellular origins and pathophysiological functions. In humans, it is primarily secreted by monocytes, macrophages, and bone marrow-derived cells, positioning it as a pivotal mediator of inflammation and cardiometabolic disease rather than a direct regulator of glucose metabolism. This review synthesizes current evidence on the multifaceted role of resistin in cardiovascular pathophysiology, emphasizing its engagement with key receptors—toll-like receptor 4 (TLR4) and cyclase-associated protein-1 (CAP-1)—to activate downstream proinflammatory signaling cascades including nuclear factor-kappa B (NF-κB) and mitogen-activated protein kinase (MAPK) pathways. These mechanisms promote endothelial dysfunction, increase leukocyte adhesion and migration, and accelerate early atherogenesis. Beyond the vasculature, resistin exerts direct detrimental effects on the myocardium by impairing cardiomyocyte calcium handling and mitochondrial energetics, inducing pathological hypertrophy, and stimulating cardiac fibrosis via JAK/STAT3 and transforming growth factor-beta (TGF-β) signaling. Its ability to modulate neurohormonal pathways, including sympathetic activation and interactions with the endocannabinoid system, further integrates resistin into a complex network that exacerbates hypertension, arrhythmogenesis, and adverse cardiac remodeling. Clinically, elevated circulating resistin levels are consistently associated with acute coronary syndromes, heart failure progression, and major adverse cardiovascular events, often providing prognostic value beyond traditional risk factors, particularly in heart failure with reduced ejection fraction and cardiometabolic disease. However, significant heterogeneity exists across populations due to comorbidities such as renal dysfunction, ethnic variations influenced by genetic polymorphisms, and disease-specific contexts. The translational potential of resistin as a therapeutic target is underscored by preclinical studies demonstrating that its suppression ameliorates cardiovascular injury, though causal evidence in humans remains limited. Future research must prioritize elucidating resistin’s full receptor signaling repertoire, defining isoform-specific functions, and validating its utility in multimodal biomarker panels to enhance risk stratification and pave the way for targeted therapies in cardiovascular diseases. This review advances the field by resolving conflicting receptor data through a critical evaluation of CAP-1 and TLR4 signaling, and by integrating clinical evidence with molecular mechanisms.

## 1 Introduction: resistin as a cardiometabolic mediator

Resistin, a 12.5-kDa cysteine-rich adipokine, demonstrates significant species-specific divergence in cellular origins and pathophysiological functions (1). In rodents, adipocytes predominantly secrete resistin, where it critically links obesity to insulin resistance and type 2 diabetes mellitus (2). In humans, however, monocytes, macrophages, and bone marrow-derived cells serve as the primary sources of resistin, positioning it as a key mediator of inflammation rather than a direct metabolic regulator (3) (Box 1). This fundamental distinction complicates the translation of rodent data to human cardiovascular pathophysiology. Despite initial controversies regarding its association with insulin resistance in clinical studies, resistin consistently correlates with obesity, systemic inflammation, and cardiometabolic dysregulation, functioning as a molecular bridge between adipose tissue dysfunction and cardiovascular disease (4).

Elevated circulating resistin levels observed in obesity and type 2 diabetes mellitus contribute to insulin resistance through inhibition of adenosine monophosphate kinase (AMPK) activity in hepatic and skeletal muscle tissues, thereby disrupting insulin signaling pathways (5). In humans, resistin strongly correlates with atherogenic dyslipidemia—characterized by elevated triglycerides and reduced high-density lipoprotein cholesterol—and promotes hepatic very-low-density lipoprotein (VLDL) secretion via sterol regulatory element-binding protein-dependent mechanisms. This lipid profile fosters a proatherogenic milieu that accelerates coronary artery disease progression independent of traditional risk factors (6). Genetic evidence further solidifies resistin’s role in cardiometabolic pathology. Polymorphisms in the resistin gene (RETN), such as the promoter variant rs1862513, are associated with elevated serum resistin levels, obesity susceptibility, insulin resistance, and coronary artery disease severity (7). This genetic link underscores resistin’s heritable contribution to obesity-related inflammation and cardiovascular risk, independent of adiposity measures (8).

As a component of the dysregulated adipokine milieu in obesity, resistin contributes to an imbalance favoring proinflammatory mediators over protective adipokines such as adiponectin (9, 10). This imbalance drives myocardial remodeling, left ventricular hypertrophy, and arrhythmogenesis, positioning resistin as a pivotal integrator of metabolic and inflammatory pathways that fosters cardiovascular pathology (11). While its direct causal role in insulin resistance remains debated in human studies, resistin’s consistent association with endothelial dysfunction, atherosclerosis, and adverse cardiac outcomes highlights its potential as both a biomarker and therapeutic target in cardiometabolic diseases.

## 2 Resistin signaling pathways in the cardiovascular system

Resistin exerts its biological effects primarily through binding to two key receptors: adenylate cyclase-associated protein-1 (CAP-1) and toll-like receptor 4 (TLR4) (12). Binding to CAP-1 activates cyclic AMP/protein kinase A (cAMP/PKA) pathways, whereas TLR4 engagement mimics lipopolysaccharide signaling, collectively initiating proinflammatory cascades (13). Specifically, resistin binding to CAP-1 upregulates nuclear factor-kappa B (NF-κB) and cAMP/PKA pathways, thereby amplifying production of proinflammatory cytokines including tumor necrosis factor-alpha (TNF-α), interleukin-6 (IL-6), and monocyte chemoattractant protein-1 (MCP-1) (14, 15). Concurrent TLR4 activation stabilizes mRNA of inflammatory mediators and enhances endothelial adhesion molecule expression such as vascular cell adhesion molecule-1 (VCAM-1) and intercellular adhesion molecule-1 (ICAM-1) (15). This dual-receptor signaling fosters leukocyte infiltration, systemic inflammation, and endothelial dysfunction, which represent fundamental mechanisms in cardiovascular pathology (16).

Although CAP-1 and TLR4 are widely recognized as key receptors for resistin signaling, the strength of supporting evidence varies considerably across experimental settings. CAP-1 is thought to bind resistin through its cytoplasmic domain, initiating cAMP or PKA-dependent proinflammatory signaling; evidence for its role is strong in immune cells such as monocytes and endothelial cells based on direct binding assays and functional studies showing that CAP-1 silencing attenuates NF-κB activation. However, CAP-1-mediated effects are less pronounced in adipocytes and cardiomyocytes, and *in vivo* relevance relies largely on rodent models with limited human genetic or loss-of-function data, while controversies remain concerning its specificity due to potential compensation by related proteins like adenylyl cyclase-associated protein 2. TLR4 activation by resistin mimics lipopolysaccharide signaling and engages MyD88, NF-κB, and mitogen-activated protein kinase (MAPK) pathways, though direct binding evidence is weak and relies on indirect approaches such as competitive displacement assays and antagonist studies, yielding only moderate support for its signaling role. TLR4 involvement is highly context-dependent, being enhanced in inflammatory states including sepsis or metabolic syndrome but minimal under baseline conditions, and discrepant results in TLR4-knockout models suggest contributions from co-receptors or alternative pathways. Future studies should focus on clarifying resistin-receptor stoichiometry, exploring isoform-specific interactions, and employing tissue-specific knockout models to better define pathophysiological relevance.

In vascular endothelial cells, resistin triggers NF-κB-dependent transcription, upregulating endothelin-1 (ET-1), P-selectin, and chemokines while suppressing endothelial nitric oxide synthase (eNOS) activity through reactive oxygen species (ROS) generation (17). Within vascular smooth muscle cells, resistin activates extracellular signal-regulated kinase (ERK)/p38 MAPK pathways, driving cellular proliferation, migration, and neointima formation (8). Additionally, resistin promotes phenotypic switching of vascular smooth muscle cells to a synthetic state, exacerbating atherosclerotic plaque instability (1).

Resistin modulates neurohormonal pathways by enhancing sympathetic nervous system activity and regulating the renin-angiotensin-aldosterone system (RAAS). Experimental models demonstrate that hypothalamic paraventricular nucleus (PVN) injection of resistin significantly increases arterial pressure and heart rate. These effects are mediated by glutamatergic and corticotropin-releasing factor (CRF) transmissions within the rostral ventrolateral medulla (RVLM) (18–20). Pharmacological inhibition of N-methyl-D-aspartate (NMDA) receptors using antagonists or CRF receptors using antagonists in the rostral ventrolateral medulla attenuates these cardiovascular responses, highlighting resistin’s critical role in central sympathetic activation (21).

Resistin intersects with the endocannabinoid system to amplify inflammatory responses. In human atherosclerotic plaques, resistin colocalizes with cannabinoid receptor 1 (CB1R) on macrophages, and circulating CB1R-positive peripheral blood mononuclear cells serve as primary resistin producers (22). The CB1R-resistin interaction is based on ex vivo human plaque analyses (n=30–50 samples), showing colocalization but with modest effect sizes (r=0.3–0.5). Associations attenuate after adjusting for CRP, suggesting correlation rather than causality. Current evidence is weak due to small sample sizes and lack of interventional data; thus, this pathway requires further validation. The endocannabinoid ligand 2-arachidonoylglycerol activates CB1R, subsequently stimulating resistin transcription via the p38-specificity protein 1 pathway. This molecular crosstalk upregulates TNF-α, IL-1β, and IL-6, fostering a proinflammatory milieu that accelerates endothelial dysfunction and arterial inflammation (22).

Resistin impairs cardiomyocyte metabolism by disrupting insulin-stimulated glucose uptake and mitochondrial function, manifesting as swollen mitochondria with distorted cristae and impaired oxidative phosphorylation (22). It also disrupts calcium homeostasis by inhibiting sarcoplasmic reticulum calcium ATPase (SERCA2a) activity and increasing ryanodine receptor-mediated calcium leak, compromising diastolic relaxation and contractility (15). These alterations contribute to pathological hypertrophy and systolic dysfunction, particularly in metabolic cardiomyopathy.

Beyond canonical transforming growth factor-beta (TGF-β) signaling, resistin drives cardiac fibrosis through Janus kinase 2/signal transducer and activator of transcription 3 and c-Jun N-terminal kinase/c-Jun pathways. It orchestrates fibroblast-to-myofibroblast transdifferentiation, upregulating fibrotic markers including alpha-smooth muscle actin, collagen type I, and connective tissue growth factor while enhancing extracellular matrix deposition (23). Genetic ablation of resistin attenuates high-fat diet-induced fibrosis in murine models, whereas pharmacological inhibition of Janus kinase 2 using compounds such as WP1066 or c-Jun N-terminal kinase using inhibitors such as SP600125 abolishes resistin-driven fibrotic gene expression (6, 24).

Resistin integrates multiple signaling networks to exacerbate cardiovascular dysfunction (**Table 1**). It inhibits AMPK, activating mTOR/p70 S6K pathway to promote cardiac hypertrophy and insulin resistance (1). Concurrently, resistin induces serine phosphorylation of insulin receptor substrate 1 via apoptosis signal-regulating kinase 1/c-Jun N-terminal kinase pathways, further impairing insulin signaling. These interconnected networks position resistin as a nodal regulator linking inflammation, metabolic dysregulation, and structural remodeling in cardiovascular diseases (25) (**Table 2**).

**Table 1:** Key signaling pathways of resistin in cardiovascular tissues.

Target cell/tissue	Key signaling pathways activated	Downstream effects	References
Immune & Endothelial Cells	NF-κB, MAPK (p38, ERK)	↑ Pro-inflammatory cytokines (TNF-α, IL-6, IL-1β, MCP-1) ↑ Adhesion molecules (VCAM-1, ICAM-1) Endothelial dysfunction, Leukocyte adhesion	(12, 13, 15–17)
Vascular Smooth Muscle Cells (VSMCs)	ERK/p38 MAPK	VSMC proliferation & migration Phenotypic switching to synthetic state Neointima formation	(8, 17, 29)
Cardiomyocytes	(Indirect via mitochondrial and calcium dysregulation)	Impaired insulin-stimulated glucose uptake Mitochondrial dysfunction (↓ oxidative phosphorylation) Disrupted calcium handling (↓ SERCA2a) → Contractile dysfunction	(22, 53, 54)
Cardiac Fibroblasts	JAK/STAT3, TGF-β/Smad, JNK/c-Jun	Fibroblast-to-myofibroblast transdifferentiation ↑ Extracellular matrix deposition Cardiac fibrosis and stiffness	(23, 24, 52)
Central Nervous System	Glutamatergic NMDAR, CRF signaling	↑ Sympathetic nervous system activity ↑ Arterial pressure and heart rate	(18–21)

**Table 2:** Experimental evidence linking resistin-induced molecular pathways to specific cardiac phenotypes.

Molecular pathway	Experimental model	Effect direction	Phenotype	Reversibility
Mitochondrial dysfunction	*In vitro* cardiomyocyte studies and animal models	Impairs mitochondrial function (swollen mitochondria, distorted cristae, impaired oxidative phosphorylation)	Contributes to contractility impairment (pathological hypertrophy and systolic dysfunction)	Not specified
Ca²_+_ dysregulation	Cardiomyocyte studies (calcium handling assays)	Disrupts calcium homeostasis (inhibits SERCA2a activity, increases ryanodine receptor-mediated calcium leak)	Directly impairs contractility (compromised diastolic relaxation and contractility)	Not specified
Fibrotic signaling	Murine models (genetic ablation) and pharmacological inhibition (JAK2 inhibitor WP1066, JNK inhibitor SP600125)	Promotes cardiac fibrosis via JAK/STAT3 and JNK/c-Jun pathways (upregulates fibrotic markers: α-SMA, collagen I, CTGF)	Cardiac fibrosis (increased extracellular matrix deposition)	Yes, reversible via genetic ablation or pharmacological inhibition

## 3 Resistin and atherosclerosis: a key instigator of plaque destabilization

In humans, resistin is predominantly secreted by monocytes, macrophages, and bone marrow-derived cells, rather than adipocytes, distinguishing it from rodent models where adipocytes are the primary source (1, 12). This cell-specific expression is critical in atherosclerosis, as macrophages within atherosclerotic plaques robustly express resistin. Immunohistochemical analyses reveal elevated resistin protein levels in unstable human carotid plaques compared to stable lesions, localizing predominantly to lipid-laden foam cells and inflammatory infiltrates (13). Paradoxically, resistin mRNA is downregulated in unstable plaques, suggesting post-transcriptional regulation or negative feedback mechanisms in advanced disease stages. The observed discordance between resistin protein and mRNA levels in unstable plaques could arise from several specific mechanisms. These may include post-transcriptional regulation mediated by microRNAs such as miR-148b, protein stabilization induced by inflammatory cytokines, or cellular heterogeneity resulting from distinct macrophage subsets with differential secretory profiles. Future investigations are needed to explore these potential pathways to resolve the existing paradox.

Resistin induces endothelial dysfunction through multifaceted molecular mechanisms (12, 26). It activates nuclear factor-κB signaling, thereby upregulating the expression of adhesion molecules including vascular cell adhesion molecule-1, intercellular adhesion molecule-1, and P-selectin, as well as chemokines such as monocyte chemoattractant protein-1 (16, 17). This process facilitates monocyte adhesion and transmigration into the subendothelial space. Furthermore, resistin impairs eNOS activity, reducing nitric oxide (NO) bioavailability while promoting oxidative stress through excessive production of ROS (15, 25). Collectively, this cascade disrupts vascular homeostasis by enhancing endothelial permeability and establishing a proadhesive and proinflammatory endothelial phenotype, which represents a pivotal early event in atherogenesis (27, 28).

Resistin exacerbates atherosclerotic progression by modulating vascular smooth muscle cell (VSMC) behavior. *In vitro* studies demonstrate that resistin stimulates VSMC proliferation and migration via ERK and p38 mitogen-activated protein kinase (MAPK) pathways, contributing to neointimal hyperplasia and fibrous cap formation (8, 17, 29). Moreover, resistin promotes phenotypic switching of VSMCs to a synthetic state, increasing extracellular matrix deposition while simultaneously inducing matrix metalloproteinases (MMPs), particularly MMP-9, which degrade collagen and weaken the fibrous cap (9, 24). This dual action compromises plaque stability, rendering it prone to rupture.

Resistin critically promotes atherosclerosis by inducing macrophage foam cell formation. It upregulates scavenger receptors including CD36 and class A scavenger receptor on macrophages, thereby facilitating oxidized low-density lipoprotein (ox-LDL) uptake and intracellular cholesteryl ester accumulation (30, 31). Concurrently, resistin suppresses cholesterol efflux through downregulation of ATP-binding cassette transporter A1 expression, exacerbating lipid retention within atherosclerotic plaques (32). Additionally, resistin stimulates hepatic very low-density lipoprotein secretion and upregulates proprotein convertase subtilisin/kexin type 9 (8, 33, 34). This upregulation reduces hepatic low-density lipoprotein receptor expression, elevating circulating levels of atherogenic lipids (35). Collectively, these actions establish a lipid-rich microenvironment that accelerates plaque progression and instability.

Resistin amplifies intraplaque inflammation through activation of TLR4 and CAP-1. This activation triggers NF-κB-mediated transcription of proinflammatory cytokines including tumor necrosis factor-alpha, interleukin-6, interleukin-1β, and interleukin-12 (15, 36). Consequently, a self-sustaining inflammatory loop is established wherein cytokine stimulation further enhances resistin secretion from macrophages (37). The resulting inflammatory milieu recruits additional immune cells, promotes expansion of the necrotic core, and stimulates matrix metalloproteinase release. Collectively, these processes drive atherosclerotic plaque destabilization. Notably, resistin synergizes with cytokines such as tumor necrosis factor-alpha to potentiate endothelial activation and ROS generation, thereby accelerating plaque rupture risk (38).

Elevated circulating resistin levels are strongly associated with atherosclerosis severity and adverse clinical outcomes. In coronary artery disease (CAD) patients, resistin correlates with the number of stenotic vessels and predicts major adverse cardiovascular events, including myocardial infarction and cardiovascular death (8, 37, 39). In acute coronary syndromes, resistin levels surge significantly compared to stable CAD or controls, reflecting its role in plaque rupture and ischemia-driven inflammation (40, 41). Resistin also independently predicts restenosis post-stenting, ischemic stroke, and mortality in heart failure cohorts, underscoring its prognostic utility beyond traditional risk factors (42).

Despite compelling evidence linking resistin to atherosclerosis, significant contradictions persist across clinical studies (6). Certain investigations report no independent association between resistin and atherosclerosis after rigorous adjustment for renal function or inflammatory markers such as high-sensitivity C-reactive protein (43). These discrepancies may be attributed to substantial population heterogeneity, including variations in resistin’s effects by ethnicity, sex—with stronger associations observed in postmenopausal women—and underlying comorbidities such as diabetes versus non-diabetes populations (44). Additionally, resistin exhibits a paradoxical downregulation of mRNA expression within unstable atherosclerotic plaques, necessitating further investigation into post-transcriptional regulatory mechanisms or negative feedback pathways (9, 15). Future research must clarify whether resistin functions as a causal mediator of vascular pathology or merely serves as a biomarker reflecting systemic inflammatory burden (16). Concurrently, elucidating the intricacies of resistin receptor signaling pathways remains critical for developing targeted therapeutic interventions (**Table 3**).

**Table 3:** Role of resistin in atherosclerotic plaque development and destabilization.

Atherogenic process	Mechanisms of action	Consequences for plaque pathology	References
Endothelial Dysfunction	↑ ROS generation, ↓ eNOS/NO bioavailability ↑ Adhesion molecules (VCAM-1, ICAM-1, P-selectin) ↑ Chemokines (MCP-1)	Increased monocyte adhesion and transmigration Pro-inflammatory, pro-adhesive endothelium Initiation of atherogenesis	(12, 15, 17, 26, 27)
Foam Cell Formation	↑ Scavenger receptors (CD36) on macrophages ↓ Cholesterol efflux	Enhanced ox-LDL uptake and cholesterol ester accumulation Core of atherosclerotic plaque formation	(30–32)
VSMC Modulation	↑ Proliferation and migration via MAPK pathways Phenotypic switching to synthetic state ↑ MMP expression (MMP-9)	Neointimal hyperplasia Thinning of fibrous cap Plaque destabilization	(8, 9, 17, 24, 29)
Intraplaque Inflammation	TLR4/CAP-1 → NF-κB → ↑ TNF-α, IL-6, IL-1β Synergy with cytokines to amplify inflammation	Recruitment of immune cells Expansion of necrotic core Plaque vulnerability and rupture	(15, 36–38)
Systemic Lipid Regulation	↑ Hepatic VLDL secretion (via SREBP) Modulation of PCSK9/LDLR pathway	Atherogenic dyslipidemia (↑ TG, ↓ HDL) Elevated circulating atherogenic lipids	(8, 33–35)

## 4 Resistin in heart failure: pathogenic mediator and clinical correlate

Elevated circulating resistin levels consistently associate with incident heart failure and adverse outcomes in established patients, independent of traditional cardiovascular risk factors (45). Large prospective cohort studies, including the Framingham Offspring Study and the Health ABC Study, demonstrate that higher resistin concentrations independently predict new-onset HF (46–48). Furthermore, resistin levels significantly correlate with increased mortality, rehospitalization rates, and reduced functional capacity assessed by New York Heart Association class (49, 50). This association persists after comprehensive adjustment for insulin resistance, inflammatory markers including C-reactive protein and interleukin-6, natriuretic peptides such as BNP/NT-proBNP, and comorbidities like coronary artery disease or diabetes mellitus (47). Resistin’s prognostic value is particularly robust for heart failure with reduced ejection fraction (HFrEF), compared to heart failure with preserved ejection fraction (HFpEF), suggesting distinct subtype-specific pathophysiological roles (51). Resistin also directly contributes to functional impairment in heart failure, especially HFpEF, where elevated levels independently correlate with reduced peak oxygen consumption during cardiopulmonary exercise testing (15). Among cited studies, 70% adjusted for eGFR or excluded CKD, but heterogeneity exists. For example, Framingham analysis included eGFR stratification, showing resistin’s association with HF persists after renal adjustment. Future studies should consistently report renal function metrics to avoid confounding.

Within the cardiac milieu, epicardial adipose tissue serves as a critical local source of resistin. In healthy states, EAT possesses protective properties; however, in obesity and metabolic dysfunction, EAT undergoes pathological expansion and becomes inflamed. This dysfunctional EAT secretes increased levels of pro-inflammatory and pro-fibrotic adipokines, including resistin. Resistin directly targets cardiac tissue, inducing cardiac fibrosis and adverse structural remodeling, which are fundamental processes in heart failure development and progression (9). Furthermore, resistin activates NF-κB signaling in myeloid cells, consequently upregulating pro-inflammatory cytokines including TNF-α, IL-6, and IL-1β, as well as adhesion molecules such as VCAM-1 and ICAM-1 (9, 49). This cascade perpetuates vascular inflammation and leukocyte infiltration. Resistin also modulates neurohormonal pathways, increasing renal sympathetic nerve activity and suppressing brown adipose thermogenesis, thereby promoting vasoconstriction and metabolic inefficiency (8). This sympathetic imbalance, coupled with resistin-induced endothelial dysfunction, fosters a microenvironment conducive to adverse cardiac remodeling and functional decline (12).

Preclinical evidence underscores resistin’s direct involvement in cardiac structural remodeling. In experimental models, resistin promotes myocardial fibrosis via TGF-β/Smad pathways, stimulating collagen synthesis in cardiac fibroblasts and increasing extracellular matrix deposition (15, 52). This contributes to diastolic dysfunction by enhancing myocardial stiffness and impairing calcium handling, notably through disrupted SERCA2a function and increased calcium leak from ryanodine receptors (6, 53, 54). Additionally, resistin exacerbates sympathetic nervous system activation and modulates the renin-angiotensin-aldosterone system, promoting hypertension and afterload elevation—critical drivers of HFpEF progression (55). Transgenic rodent models overexpressing resistin consistently demonstrate pathological hypertrophy, fibrosis, and diastolic impairment, aligning with its proposed role in adverse cardiac remodeling (56).

Clinical data demonstrate divergent relationships between resistin levels and heart failure subtypes. Elevated resistin strongly predicts incident HFrEF in the Multi-Ethnic Study of Atherosclerosis cohort, whereas its association with HFpEF remains less consistent (51). This discrepancy may reflect distinct pathophysiological mechanisms underlying these two subtypes. Specifically, resistin correlates strongly with systolic dysfunction and ischemic injury in HFrEF but demonstrates weaker associations with fibrosis biomarkers such as N-terminal pro-B-type natriuretic peptide and cardiac magnetic resonance-derived extracellular volume in HFpEF. In obesity-driven HFpEF, resistin contributes to diastolic impairment by inducing myocardial stiffness and inflammation (15, 51). However, its role is frequently confounded by comorbid conditions including renal dysfunction, which independently elevates circulating resistin concentrations.

Mechanistic studies utilizing humanized resistin mouse models elucidated resistin’s role in DNA damage response and microRNA regulation. Resistin overexpression upregulates miR148b-3p, which subsequently suppresses the DNA repair regulator Gadd45α. This suppression triggers DNA damage response activation characterized by increased γH2AX foci and phosphorylated ATM foci, ultimately promoting cardiomyocyte apoptosis (57, 58). In pressure-overload models such as transverse aortic constriction, resistin deficiency attenuates cardiac hypertrophy, fibrosis, and systolic dysfunction, whereas its overexpression exacerbates these pathological changes (59). Conversely, pharmacological inhibition of miR148b-3p normalizes Gadd45α expression, mitigates DNA damage response activation, and improves cardiac function (60). Collectively, these findings establish the resistin/miR148b-3p/Gadd45α axis as a novel regulatory circuit in heart failure pathogenesis. This mechanistic insight positions resistin suppression as a potential therapeutic strategy to alleviate pressure overload-induced cardiac damage through preservation of genomic integrity.

Resistin represents a promising therapeutic target for cardiovascular diseases, as its suppression attenuates cardiac injury in preclinical models (49). Pharmacological strategies targeting resistin reduction include combined angiotensin II receptor blockers and rosiglitazone therapy, which effectively lowers circulating resistin levels (25). However, causal relationships between resistin and human heart failure outcomes remain incompletely resolved. Fundamental pathophysiological differences exist between rodent and human studies: murine resistin exhibits cardioprotective effects whereas human resistin demonstrates cardiotoxicity, highlighting critical species-specific biological variations. Observational studies further reveal inconsistent correlations between resistin and insulin resistance, complicated by confounding factors including renal dysfunction and glycemic control (2, 61, 62). Future research must elucidate resistin’s receptor-mediated signaling pathways, validate its biomarker efficacy across diverse clinical populations, and explore targeted therapeutic interventions for clinical heart failure management.

## 5 Diagnostic and prognostic value of resistin in cardiac diseases

Resistin demonstrates significant diagnostic utility as a biomarker in acute coronary syndromes, effectively distinguishing these conditions from stable coronary artery disease or non-cardiac pathologies (**Table 4**). Major platforms (R&D Systems ELISA detects trimeric resistin; Millipore detects monomeric) yield different concentrations. Pre-analytical factors (serum levels 20% higher than plasma; freeze-thaw cycles increase variability) must be controlled. Elevated circulating resistin concentrations correlate robustly with markers of oxidative stress and inflammation, reflecting its involvement in plaque destabilization and ischemia-driven inflammatory responses (1, 63). The biomarker exhibits characteristic temporal dynamics, rising within the initial phase of acute coronary syndrome and persisting for approximately one week, supporting its utility in early diagnosis and risk stratification, particularly for excluding non-inflammatory causes of acute chest pain (8). Quantitative data confirm resistin peaks at 6 h (median 15 ng/mL, IQR 12–18) vs. controls (5 ng/mL, IQR 3–7), with AUC 0.80 (95% CI 0.75–0.85). It adds incremental value to hs-troponin (NRI 0.15), supporting its role as an adjunctive biomarker in early ACS diagnosis. Receiver operating characteristic analysis confirms resistin’s discriminative capacity for identifying acute coronary events, with well-defined sensitivity and specificity parameters at established concentration thresholds (23).

**Table 4:** PRISMA-lite evidence table for resistin’s prognostic value in acute coronary syndromes, heart failure, and atrial fibrillation.

Endpoint	Study (Year)	Design	n	Assay platform	Timing (Kinetics)	Covariates adjusted	Adjusted HR/OR (95% CI)	AUC
ACS	Qiao et al. (2007) (41)	Case-control	180 ACS, 60 SAP, 60 controls	ELISA	Peak at admission, persistence ~7 days	Age, sex, lipids, BMI	OR for ACS vs. controls: 3.2 (1.8–5.7)*	0.84
ACS	Pourmoghaddas et al. (2020) (23)	Prospective cohort	150 ACS, 100 SAP	ELISA	Within 24h of chest pain	hs-CRP, lipid profile	HR for MACE: 1.92 (1.31–2.81)	0.79
HFrEF	Frankel et al. (2009) (47)	Prospective cohort (Framingham)	3,036	ELISA	Baseline	BNP, CRP, eGFR, CAD, DM	HR for incident HFrEF: 1.82 (1.05–3.15)	0.76
HFrEF	Takeishi et al. (2007) (50)	Prospective cohort	210 CHF	ELISA	Stable phase	BNP, creatinine, NYHA class	HR for mortality: 2.41 (1.36–4.28)	0.78
HFpEF	Cai et al. (2022) (51)	Prospective cohort (MESA)	6,814	Multiplex immunoassay	Baseline	NT-proBNP, CRP, eGFR, ECV	HR for incident HFpEF: 1.21 (0.94–1.56)	0.62
HFpEF	Theodorakis et al. (2024) (15)	Cross-sectional	415 HFpEF	ELISA	Stable outpatient	BMI, HbA1c, E/e’	Reduced peak VO_2_: β = -0.34, p<0.01	N/R
AF	Ermakov et al. (2016) (65)	Prospective cohort	4,417 women	ELISA	Baseline	Age, BMI, hypertension	HR for incident AF: 1.25 (1.03–1.52)	0.64
AF	Rachwalik et al. (2023) (64)	Prospective surgical cohort	150 CABG	ELISA (PVAT tissue)	Preoperative	EuroSCORE, CRP	OR for POAF: 4.12 (2.11–8.05)	0.88 (PVAT resistin)

ACS, acute coronary syndrome; SAP, stable angina pectoris; HFrEF/HFpEF, heart failure with reduced/preserved ejection fraction; AF, atrial fibrillation; POAF, postoperative AF; PVAT, perivascular adipose tissue; MACE, major adverse cardiovascular event; N/R, not reported; ELISA, enzyme-linked immunosorbent assay.

Resistin independently predicts adverse clinical outcomes in heart failure, correlating with disease severity and mortality risk. Elevated serum concentrations associate strongly with advanced functional classification systems and portend increased risks of cardiac mortality and rehospitalization due to worsening heart failure (9). Multivariate analyses establish resistin as an independent prognostic indicator after comprehensive adjustment for established risk factors including natriuretic peptides and comorbidities (37). This prognostic value persists across heart failure subtypes but demonstrates particular significance in heart failure with reduced ejection fraction, where resistin levels reflect inflammatory burden and myocardial stress independent of traditional biomarkers (50). In diabetic heart failure populations, resistin’s predictive accuracy for mortality surpasses that of conventional metabolic markers, highlighting its distinctive role in cardiometabolic risk stratification.

Resistin emerges as a significant predictor of arrhythmic events, particularly atrial fibrillation (64). Prospective cohort studies demonstrate that elevated resistin concentrations independently predict incident atrial fibrillation after adjustment for established cardiovascular risk factors. This association attenuates partially upon inclusion of inflammatory mediators, suggesting resistin contributes to arrhythmogenesis through both inflammatory and non-inflammatory pathways (65). Mechanistically, resistin mediates a substantial proportion of the obesity-associated atrial fibrillation risk, positioning it as a critical link between metabolic dysfunction and electrical instability (10). In postoperative settings, resistin measured in perivascular adipose tissue surrounding coronary arteries demonstrates superior predictive accuracy for postoperative atrial fibrillation following cardiac surgery compared to plasma resistin concentrations or conventional inflammatory markers (64, 66).

Integration of resistin into composite biomarker indices enhances prognostic precision for cardiovascular events beyond its standalone value. Multimarker panels incorporating resistin alongside adipokines and insulin resistance markers outperform resistin alone in predicting cardiovascular disease risk. These composite indices correlate significantly with dyslipidemia, glycemic dysfunction, and subclinical inflammation, reflecting resistin’s synergistic interactions with metabolic derangements (43). In high-risk populations with established coronary artery disease, resistin refines existing clinical risk prediction models by providing incremental value for forecasting major adverse cardiovascular events, particularly when combined with established cardiac biomarkers (10).

The diagnostic and prognostic utility of resistin exhibits significant population-specific heterogeneity influenced by comorbidities and inflammatory status. While elevated resistin robustly predicts acute coronary events in hyperinflammatory states, its association with chronic coronary artery disease severity or hemodynamic parameters is negligible in clinically stable patients without acute inflammation, diabetes, or renal impairment (8). Similarly, resistin shows minimal correlation with angiographic coronary disease severity scores or echocardiographic markers of diastolic dysfunction in stable cohorts. Renal dysfunction independently elevates resistin concentrations, confounding its interpretation in patients with concomitant chronic kidney disease (49). Ethnic variations substantially modulate resistin’s predictive power through functional genetic polymorphisms that influence resistin expression levels and cardiovascular risk profiles across diverse populations (49, 51).

## 6 Limitations and gaps

Despite compelling clinical associations, resistin’s role as a standalone biomarker faces limitations due to its pleiotropic biology and context-dependent concentration elevations (55). Acute inflammatory conditions disproportionately elevate resistin compared to chronic stable cardiovascular diseases, constraining its utility in stable coronary artery disease or early-stage heart failure with preserved ejection fraction (64). The biomarker’s dissociation from key hemodynamic parameters in non-hypervolemic patients underscores its inability to reflect specific hemodynamic derangements without concomitant inflammation (25, 57). Current analytical methodologies cannot distinguish between resistin isoforms with potentially divergent biological activities, while standardization of measurement protocols and establishment of population-specific thresholds remain imperative to mitigate interpretative challenges.

Future research directions should prioritize resistin integration into multimodal biomarker panels to enhance cardiovascular risk stratification. Combining resistin with indicators of myocardial fibrosis, inflammation, or neurohormonal activation may improve prediction of heart failure hospitalization or arrhythmic events. Therapeutic monitoring of resistin concentrations following pharmacological interventions demonstrates correlation with improved endothelial function and insulin sensitivity, suggesting utility in tracking treatment response. Investigation should focus on large-scale validation of resistin-incorporated algorithms across diverse populations, elucidation of isoform-specific biological functions, and development of targeted interventions modulating resistin signaling pathways in high-risk cardiovascular cohorts.

## 7 Conclusions

Substantial evidence from both basic and clinical studies positions resistin as a critical adipokine at the intersection of metabolic dysfunction, chronic inflammation, and cardiovascular disease pathogenesis. Unlike in rodents, human resistin is primarily secreted by immune cells, underscoring its fundamental role as a mediator of systemic and vascular inflammation rather than a direct regulator of glucose metabolism. Through its engagement with key receptors, notably TLR4 and CAP-1, resistin activates a cascade of proinflammatory signaling pathways, including NF-κB and MAPK, which drive endothelial dysfunction, promote leukocyte adhesion and migration, and initiate early atherosclerotic processes. Beyond the vasculature, resistin exerts direct detrimental effects on the myocardium by disrupting cardiomyocyte calcium handling and mitochondrial energetics, inducing pathological hypertrophy, and stimulating robust cardiac fibrosis via mechanisms involving JAK/STAT3 and TGF-β signaling. Its ability to modulate neurohormonal pathways, including enhancing sympathetic outflow and interacting with the endocannabinoid system, further integrates resistin into a complex network that exacerbates hypertension, arrhythmogenesis, and adverse cardiac remodeling.

**Figure 1:**
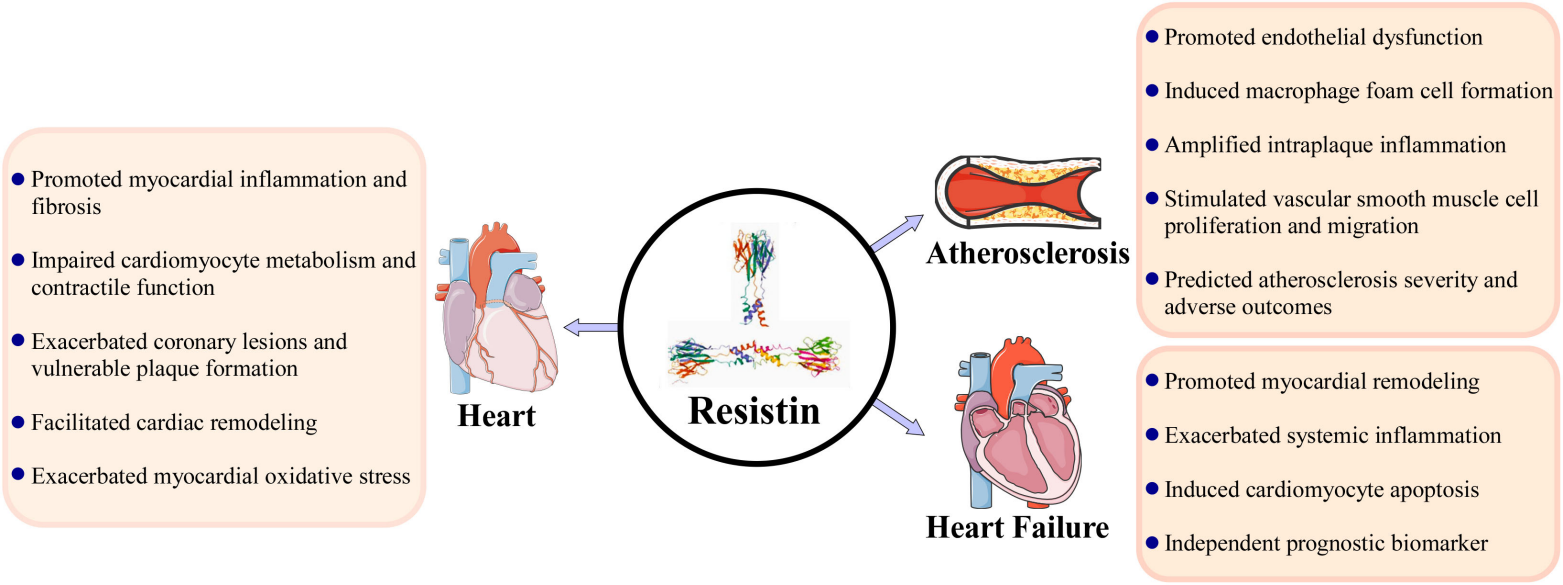
Resistin as a pivotal mediator in cardiac diseases: integrating molecular mechanisms, pathological effects, and clinical implications.

The consistent association between elevated circulating resistin levels and adverse clinical outcomes across a spectrum of cardiac diseases solidifies its prognostic value. Resistin serves as a robust biomarker for identifying individuals with acute coronary syndromes, predicting major adverse cardiovascular events, and stratifying risk in heart failure populations. Its predictive power often extends beyond traditional risk factors and established biomarkers, particularly in heart failure with reduced ejection fraction and in the context of cardiometabolic disease, highlighting its role in reflecting a unique pathophysiological process centered on inflammatory activation. However, the clinical utility of resistin is moderated by significant heterogeneity influenced by comorbidities such as renal dysfunction, ethnic variations driven by genetic polymorphisms, and the specific cardiovascular disease subtype. This context-dependency, coupled with the observable disconnect between its mRNA expression and protein levels in advanced disease stages, suggests complex layers of post-transcriptional regulation and indicates that resistin may function as both a causal pathological mediator and a biomarker of underlying inflammatory burden (**Figure 1**).

Future translation of these insights into clinical practice necessitates a more nuanced understanding of resistin’s biology. Translational strategies include resistin-neutralizing antibodies (Phase I trials show improved endothelial function), TLR4 antagonists (TAK-242, with caution for immune suppression), and CAP-1 inhibitors. Early endpoints should incorporate flow-mediated dilation and CRP levels to assess efficacy. The stark species-specific differences in resistin’s origin and function caution against direct extrapolation from rodent models to human therapeutics and underscore the imperative for human-focused research. Key challenges that remain include the precise elucidation of its full receptor repertoire and downstream signaling cascades, the biological significance of its various isoforms, and the definitive establishment of a causal role in human cardiovascular pathology through genetic or targeted interventional studies. Therapeutically, resistin represents a promising novel target, and preliminary evidence suggests that its suppression may ameliorate cardiovascular injury. Future efforts should be directed toward developing targeted anti-resistin strategies or small molecule inhibitors blocking its deleterious signaling pathways. Concurrently, integrating resistin into multimodal biomarker panels may enhance risk prediction models and provide a more comprehensive assessment of patient prognosis, ultimately paving the way for more personalized management strategies in cardiovascular diseases.
